# How effective is the early support program *Babylotse-Plus* for psychosocially burdened mothers and their infants? A comparative intervention study

**DOI:** 10.1186/s40748-019-0109-5

**Published:** 2019-08-22

**Authors:** Christine Klapp, Silvia Fisch, Theresa Keller, Ulrike Stasun, Nurina Nazmy, Cynthia Hohmann, Larry Hinkson, Wolfgang Henrich, Karl E. Bergmann, Renate L. Bergmann, Thomas Keil

**Affiliations:** 1 Department of Obstetrics, Charité – Universitätsmedizin Berlin, 10098 Berlin, Germany; 2 Department of Neonatology, Charité – Universitätsmedizin Berlin, 10098 Berlin, Germany; 3 Institute for Social Medicine, Epidemiology and Health Economics, Charité – Universitätsmedizin Berlin, 10098 Berlin, Germany; 4 Institute of Clinical Epidemiology and Biometry, University of Wuerzburg, 97070 Wuerzburg, Germany; 5 Institute for Health Resort Medicine and Health Promotion, Bavarian Health and Food Safety Authority, 97688 Bad Kissingen, Germany

**Keywords:** Child welfare, Early support, Infants, Intervention study, Maternal depression, Postnatal depression, Parenting Stress Index, Prevention, Psychosocial risk

## Abstract

**Objectives:**

Our aim was to examine the effects of an early perinatal prevention program offered to mothers and families suffering from significant psychosocial burden.

**Methods:**

All mothers giving birth in a Berlin university hospital during Jan-Aug 2013 were screened with a standardized 27-item questionnaire by trained staff. Mothers with a screening-score ≥ 3, who were not enrolled in other public support programs, were defined as psychosocially burdened. They received a detailed needs assessment and were followed up with counseling. When necessary, affected mothers were voluntarily guided through to specialized ‘early support’ institutions during the 12-month-intervention period. The historical control group (care-as-usual) consisted of children born at the same hospital the year before.

At 12 months postnatally, we interviewed mothers in both groups to assess their stress burden and coping skills by Parenting Stress Index and assessed the current childcare condition. Differences between the groups were compared by multivariable logistic regression analyses adjusting for potential confounders.

**Results:**

The intervention group and the control group included 225 and 157 families, respectively. After 12-months, mothers in the ‘early support’ intervention group had significantly less often depression (adjusted odds ratio 0.25, 95%-confidence interval 0.07–0.94), less often a disturbed relationship with the parenting partner (0.34, 0.10–1.14) and reported reduced stress due to the child’s demands (0.40, 0.15–1.10) compared to the control group. Childcare indicators did not differ between the 2 groups.

**Conclusions:**

In mothers at high psychosocial risk, the ‘early support’ intervention program *Babylotse-Plus* seemed to reduce the occurrence of depression and several stress indicators in the first postnatal year.

## Significance

Postnatal maternal depression and parenting deficits could be precursors of child abuse and neglect; however previous prevention research efforts have been lacking good quality intervention studies.

The present article summarizes methods and promising findings from a stringently conducted comparative intervention study in Berlin over a 12-month period after birth. Mothers who were at psychosocial risk received an individually tailored support program connecting to various locally based, supporting institutions. Compared to the control group receiving care-as-usual mothers in the intervention group were considerably less often depressed and showed further positive attitudes towards their child and parenting partner 12 months after birth.

## Introduction

Two child deaths occurred because of child maltreatment (physical abuse and neglect) every week in Germany at the beginning of this century and almost 3500 deaths have been reported annually in industrialized countries [1]. Child maltreatment by physical, emotional or sexual abuse is a global public health problem that not only contributes to child mortality but substantially to childhood morbidity including impairments of cognitive function, emotional and social development [2, 3]. Among the long-lasting health consequences that persist into adulthood are increased risks of chronic diseases, e.g. mental illnesses and obesity, as well as criminal and risky sexual behavior [4]. The global cost of child maltreatment has been estimated to be US$ 3.6 trillion, or 4.2% of the world’s gross domestic product, considering strains on health, social and welfare services as well as criminal justice systems [5, 6].

Programs such as home visiting, parent education and sexual abuse prevention programs were single interventions with the potential to prevent child maltreatment, according to a comprehensive evaluation of meta-analyses and reviews. Multi-component interventions combining services such as family support, parenting skills, and childcare were judged as possibly effective, but the evidence level has been low due to methodological weaknesses in previous research efforts [7]. Policymaking to prevent child abuse in many countries is still not being based on reliable observational and interventional research data. The Global Status Report on Violence Prevention 2014 by the World’s Health Organization showed that with regard to child maltreatment, 71% of countries worldwide have national action plans but only 41% collect national population-based data on this issue [8].

Depression and parenting deficits could be precursors of child abuse and neglect, so early detection and secondary or even primary prevention are crucial [9]. Postnatal depression may also be an early life stressor given known associations with lower levels of sensitive, responsive care which are needed for the development of infant health attachment relationships, emotional regulation skills, interpersonal skills and stress response mechanisms [10].

Thus, there is a pressing need to reduce the high burden of serious and long-term consequences from child abuse and neglect by developing better preventative strategies starting as early as infancy. In Germany, the National Centre on Early Prevention has been set up, by the Ministry of Family Affairs, which specifically supports early life intervention research such as our ‘Babylotse-Plus’ project. The aim of this comparative intervention study was to examine the preventative effects of a 12-month long ‘early support’ program, starting soon after birth, for severe psychosocially burdened families compared to the routine postnatal care.

## Methods

### Study design, setting and population

For all mothers who gave birth at the obstetric department of the Charité University Hospital in Berlin, Germany, between January and August 2013 (intervention group), the short *Babylotse-Plus* screening form with 27 items was completed by midwives and nursing staff to assess the overall psychosocial burden of the family. The items of the screening form included addiction, domestic violence, parental mental conditions and/or severe physical handicaps, problems with partner, 4 or more children, 2 or more children under 5 years, too few visits of the antenatal care program, smoking in pregnancy, low birth weight, preterm birth, complications at delivery, maternal age, multiple births, migration status, perception of the staff regarding mother-child bonding or lack of family support after birth, etc. At the end of this assessment, which took about 5 min, a sum score was calculated. Mothers/families with scores of 3 or more points were defined as being “likely at risk”. In a recent evaluation of the diagnostic accuracy, the *Babylotse-Plus* screening form using a cut-off at 3 performed very well in identifying families at high psychosocial risk [11].

#### The intervention group

mothers/families who were screened to be “likely at risk” received the intervention, a one-hour long comprehensive standardized interview by a trained social worker, if possible together with the child’s father, to assess in detail stress factors and possible family resources. If the comprehensive interview confirmed a high psychosocial burden, then in cooperation with the parents, an individually tailored support program connecting to various locally based, supporting institutions was offered. The included families were followed up with a telephone call at 3–4 weeks and at 3–4 months with further questioning, addressing issues such as: if the family had engaged with the support, if the problems were improved and/or if new problems had emerged. Participation in the present study was based on informed written consent and included follow-up contact after 12 months to evaluate the intervention program.

#### The historical control group

We included retrospectively mothers of children who were born at the same hospital in 2012, i.e. before the early prevention program with the specially trained social workers was implemented. The screening therefore was conducted retrospectively using the same screening form as for the intervention group. We evaluated all available hospital records including the standard mother’s pass (“Mutterpass”), postnatal course and perinatal files for determining the psychosocial risk score.

The historical control group received the full range of care that was standard at that time in our hospital. Therefore, if families with a presumed psychosocial risk had come to the attention of the doctors, nursing staff or midwives they would have received support and individual recommendations for further help. Thus, mothers/parents of the historical control group had received “service as usual”, i.e. the high standard of care in place before the additional on-top intervention with the specially trained social workers. Long-time before our program started, “service as usual” has been including the standard *Well Baby Program* in Germany with five appointments during the first year of live. It is being utilized by over 90% of the families and includes routine counseling before discharge, recommendations for an early visit to a pediatric or family doctor’s practice or an outreach clinic, and recommendation for claiming a midwife-service for 10 days - but without any systematic approach or direct support for implementation [12–14]. In case of strong indications for psychosocial problems, parents received referrals as to where to obtain support but without further implementation follow up.

Ethical approval for the study was received by the hospital’s review board (ref. no. EA2/088/12).

### Description of intervention

The specially trained social workers guided all mothers, who were likely to be at high psychosocial risk and not enrolled in other public social support programs (such as mandatory cooperation with Social Services, public healthcare or youth welfare) to special institutions offering voluntary ‘Early Support’ (“Frühe Hilfen”) during the first three years of life. These included arranging referrals or implementing access to visiting parent-counselors during pregnancy, according to the individual needs. The social workers would also give support with filling applications, attending perinatal classes, domestic midwife support for up to one year (“family-midwife”), early home visits by social workers (public health service for primigravidas), placement at an agency for parent-counseling, debt counseling, and access to a drop-in centre for further questions.

### Outcome assessment of maternal factors at 12 months

In both the intervention and the control groups, the mothers were invited and interviewed face-by-face as soon as the children reached the age of 12 months. We used the Eltern-Belastungs-Inventar (EBI), which is the validated German version of the Parenting Stress Index (PSI), with 48 parent-reported items to assess mental health, coping skills, social factors and importantly, the perceived parenting competences and influencing factors due to child characteristics and behavior [15, 16]. In addition, specific indicators of the current care conditions of the child were assessed (for details see below).

The outcome ‘Maternal stress’ was evaluated by the “Eltern Belastungs Index (EBI)”, which is the German version of the validated and widely used Parenting Stress Index (PSI) [15, 16]. The ‘Parent Section’ assessed self-reported maternal stress related to parental characteristics consisting of seven subscales: Doubt in Parenting Competence, Social Isolation, Attachment, Health, Role Restriction, Depression, and Partner Relationship. The ‘Children’s Section’ assessed parent-reported stress factors related to the child’s characteristics and behavior consisting of the following 5 subscales: adaptability, demand, mood, hyperactivity and acceptability. In total, there are 48 items, belonging to 12 different subscales. The raw values of each item were summed up to a main result and matched to a T-standard (for the total sum score and the sub-domains) or a Standard Nine (“Stanine”) value from 1 to 9 for the subscales within the sub-domains, to achieve a normal distribution. A group of 538 mothers with young children was used for standardization in the development process of the EBI.

In our analyses, the total EBI Sum Score, the parent domain scale and the children’s domain scale were each used as a binary outcome, considering T-values ≥60 as “high stress”. To evaluate specific sources of stress each subscale was used as binary outcome, considering Stanine-values ≥7 as stress.

### Outcome assessment of child care factors at 12 months

Child care status was evaluated using items indicating the current care condition of the child: nutritional status, condition of clothes, appropriate clothing according to the weather, appropriate clothing size, condition of hair, condition of skin in general and specifically in the diaper area, and condition of the finger- and toenails. For each item, the response categories were ‘normal’ and ‘reduced/neglected’, and ‘excessive’ for nutritional status and clothing size. The examination by specially trained pediatric nurses included surveillance for signs of neglect or abuse. The evaluation included first a comparison of ‘normal’ in all categories versus not normal in at least one child care category, and then a comparison of each item separately.

### Statistical methods

Descriptive statistics are presented in tabular format: means and standard deviations were calculated for variables with continuous scaled data, whereas absolute and relative frequencies for categorically scaled data. Missing values are not included in the derivation of percentages.

To investigate differences between the intervention and control group the Mann-Whitney-U Test was performed on continuous scaled variables and the Fisher's exact test for categorically scaled variables. Potential effects on the outcome (total and sub scores of maternal stress) were analyzed by univariable logistic regression analyses using the group variable as influencing variable; results were presented as unadjusted odds ratios (OR) and 95%-confidence intervals (95%-CI). Subsequently, multivariable logistic regression analyses including potential confounders were calculated and results presented as adjusted odds ratios (aOR) with 95%-CIs.

In the multivariable analyses, we considered those variables as possible confounders that were assumed to be associated with outcome and intervention and showed considerable differences between intervention and control group at baseline: (i) Maternal sickness as a potential barrier for childcare (yes/no); (ii) premature birth (yes/no); (iii) 2 or more small children aged < 5 years at home (yes/no); (iv) smoking during pregnancy (yes/no); and (v) distress by economic problems (yes/no).

We consider our analytic approach as explorative rather than strictly hypothesis testing and did not adjust for multiple testing. Therefore, results should be interpreted carefully. All analyses were performed with *Statistical Analysis System* (SAS) version 9.4 (SAS Institute, Cary, North Carolina, USA), under Windows operating system.

## Results

### Basic characteristics of study population

**Fig. 1:**
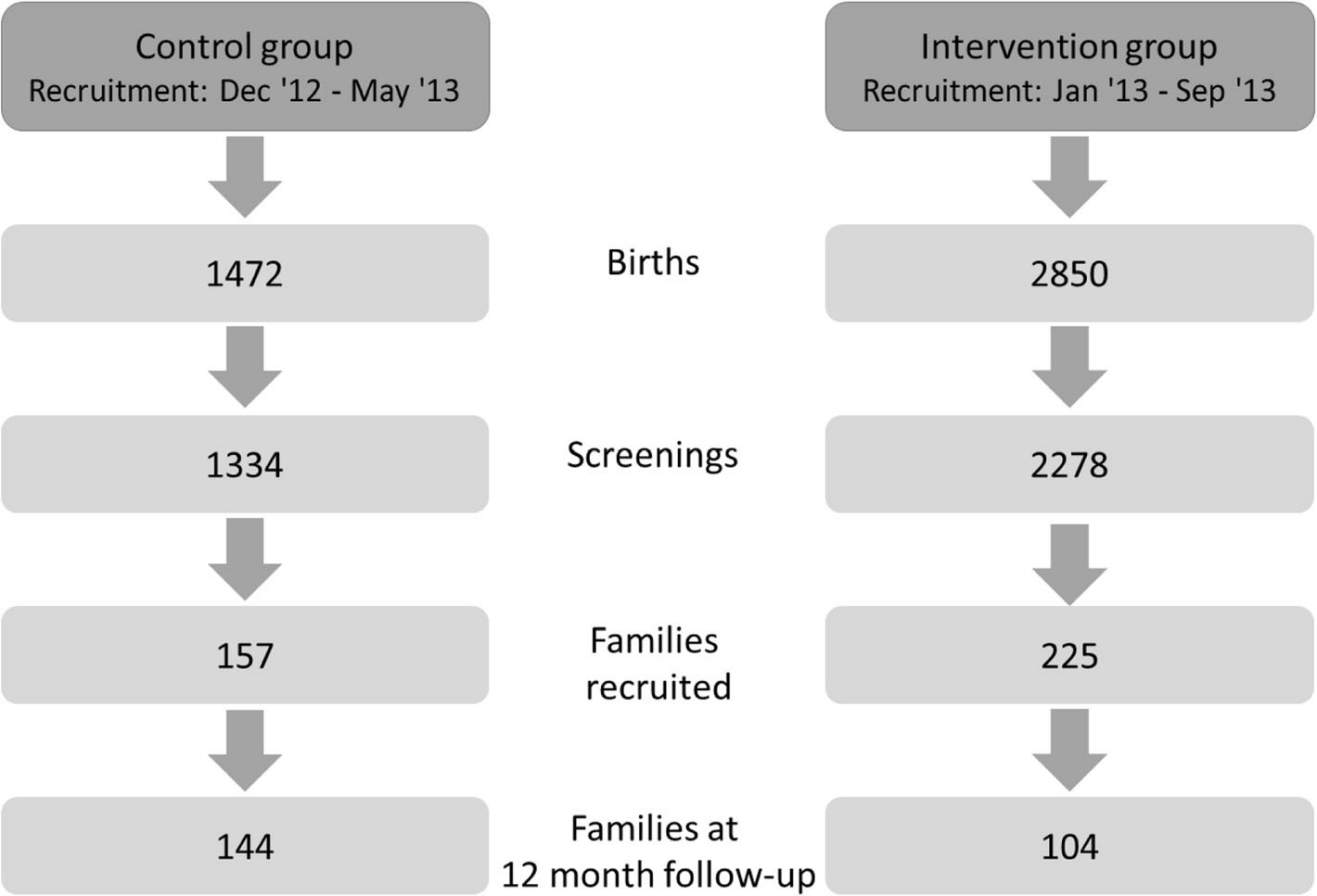
Flow chart regarding number of families in historical control- and intervention group

Out of 2278 screened families for possible recruitment in the intervention group and 1334 in the control group, we included 225 in the intervention group, 157 respectively for control (Fig. 1). There were several differences at baseline between the two comparison groups. The 225 mothers (55% had female newborns) in the intervention group had more multiple births, a late first prenatal care visit (after the 20th week of pregnancy), smoked more often during pregnancy and had more often a migration background. The 157 mothers (47% had female newborns) included in the control group had more premature babies, more often 2 or more young children at home and suffered more often from a medical condition that could be considered a barrier for childcare. They had also more often considerable economic problems (Table 1). There was no considerable difference between the risk scores of the two study groups among the children who participated versus those who did not participate in the 12-month follow-up (Table 2).

**Table 1:** Baseline characteristics of participants, by study group

	Intervention *N* = 225	%	Control *N* = 157	%	*p*-Value
Newborn’s sex					
Female	124	55.1	75	47.8	
Male	100	44.4	82	52.2	0.173
Intersex	1	0.4	0	0	
Birth weight < 3rd percentile	13	6.8	8	5.3	0.653
Multiple births	28	14.0	8	5.3	0.008
Premature delivery	29	14.9	38	24.8	0.028
Maternal age < 18 years	0	0.0	2	1.3	0.175
Maternal age < 21 years	33	15.3	15	9.7	0.120
2 or more children aged < 5 years (at home)	60	27.0	59	39.1	0.017
More than 4 children	23	10.6	10	6.5	0.197
First prenatal care visit after 20 weeks of pregnancy week	16	7.4	1	0.7	0.002
Smoking during pregnancy	69	31.1	34	21.9	0.017
Alcohol consumption during pregnancy	0	0.0	4	2.6	0.036
Indication of other addiction problems	1	0.5	1	0.6	0.763
Diagnosed psychiatric illness	16	7.1	11	7.2	1.000
Depression	7	3.1	5	3.2	1.000
Psychosis	1	0.4	0	0.0	1.000
Borderline	2	0.9	1	0.6	1.000
Other	9	4.0	5	3.2	0.704
Maternal sickness or handicap that is a barrier for childcare	9	4.3	49	32.2	< 0.001
Psychological distress through unwanted/repressed pregnancy	11	5.4	2	4.8	1.000
Psychological distress by ill / disabled family members	15	7.3	4	9.1	0.754
Psychological distress caused by problems with the partner relationship	18	8.9	2	4.9	0.542
Psychological distress caused by lack of coping with everyday life	29	14.1	6	14.3	1.000
Indication of domestic violence	6	3.0	0	0.0	1.000
Distress by integration problems	23	11.3	2	4.2	0.183
Distress by economic problems	45	21.7	23	42.6	0.003
Past / existing links to supporting institutions	14	7.3	1	2.4	0.319
Migration Background	132	60.0	69	45.7	0.008

**Table 2:** Baseline psychosocial burden risk score comparing participants *without* and those *with* 12-month follow-up

Participants without 12-month follow up				Participants with 12-month follow up		
Group	N	Median	Interquartile Range	N	Median	Interquartile Range
Intervention group (*N* = 225)	121	5	3.0–7.0	104	5	4.0–7.0
Control group (*N* = 157)	13	4	3.0–8.0	144	5	3.0–6.5

After the comprehensive 1-h interview assessment, the mothers in the intervention group received the following services: (i) early visit by the child and youth health services of the local health authority (39%); (ii) postnatal midwife service including at least 10 visits (39%); (iii) domestic and family help (14%); (iv) special parent support (administrative help etc) by the youth office (11%); (v) district family centre (4%); visits from volunteers of charity organizations (4%); and family midwife service for 1 year (3%).

### Maternal outcomes

At the end of the study, when the children were 12 months of age, the overall Parenting Stress Index (EBI in German) and most of its sub-scores were rather similar between the two groups after adjusting for potential confounders or favored slightly the intervention group. After one year, mothers in the ‘early support’ program were considerably less often depressed compared to mothers in the control group. Furthermore, they had a better relationship with their parenting partner, had less often stress due to the child’s demands, and their parental competence was higher although these differences failed to reach the conventional level of statistical significance after adjusting for potential confounders (Table 3).

**Table 3:** Maternal outcome (self-reported ‘parenting stress’) assessed by EBI-index* at the child’s age of 1 year, comparing intervention group vs historical control group (as reference group)

Outcome Parameters EBI sum score and subscores	N Inter-vention	N Control	Crude Odds Ratio ‘early support‘vs. control group (as reference)	95%-Confidence Interval	Adjusted Odds Ratio^a^ ‘early support‘vs control group (reference)	95%-Confidence Interval
EBI sum score (T-value ≥60)	21	35	0.82	0.44–1.52	0.74	0.29–1.92
EBI Subscale Parents total (T-value ≥60)	23	36	0.83	0.46–1.52	0.57	0.23–1.43
EBI Subscale Children total (T-value≥60)	22	28	1.09	0.58–2.04	0.91	0.35–2.35
Subscale Parents						
Depression (yes vs no)	78	127	0.37	0.18–0.74	0.25	0.07–0.94
Lack of mother-child attachment (yes vs no)	59	74	1.22	0.74–2.03	1.18	0.52–2.68
Social isolation (yes vs no)	79	102	1.27	0.71–2.26	1.32	0.55–3.20
Parental incompetence (yes vs no)	53	95	0.51	0.31–0.86	0.47	0.20–1.08
Health problems (yes vs no)	74	106	0.86	0.49–1.52	1.06	0.45–2.46
Personal restrictions (yes vs no)	79	115	0.77	0.42–1.42	0.85	0.32–2.23
Disturbed relationship with parenting partner (yes vs no)	72	114	0.61	0.32–1.14	0.34	0.10–1.14
Subscale Parental Stress due to Child’s...						
Demands (yes vs no)	69	117	0.42	0.23–0.76	0.40	0.15–1.10
Hyperactivity (yes vs no)	84	114	1.07	0.57–2.02	0.69	0.25–1.90
Mood (yes vs no)	49	75	0.81	0.49–1.34	0.61	0.27–1.38
Acceptability (yes vs no)	54	74	1.01	0.61–1.67	0.91	0.39–2.12
Adaptability (yes vs no)	77	108	0.92	0.52–1.65	0.54	0.21–1.40

* EBI - German version of the self-reported Parenting Stress Index (PSI)

^a^ logistic regression model adjusted for: (i) sickness of mother that is a hindrance to childcare; (ii) premature birth; (iii) 2 or more small children under age 5 at home (iv) smoking during pregnancy; and (v) distress by economic problems

### Childcare outcomes

There were no considerable differences between the children in the ‘early support’ program compared to the control group in terms of childcare condition; both for the overall assessment as well as for single factors such as nutritional status, clothing or hair/skin related assessments (Table 4).

**Table 4:** Current condition of childcare at the age of 12 months, by study group

Child care condition	‘Early support‘intervention group (*N* = 104) n	%	Control group (*N* = 144) n	%	p-value^a^
Not normal in one or more of the single care conditions	10	9.6	14	9.7	1.000
Nutritional status					
normal	95	91.3	129	90.8	
overweight	3	2.9	7	4.9	0.655
reduced / neglected	6	5.8	6	4.2	
Cloths overall					
normal	102	98.1	143	100	0.176
reduced / neglected	2	1.9	0	0.0	
Clothing - appropriate to weather					
normal	103	99.0	143	100	0.421
reduced / neglected	1	1.0	0	0.0	
Size of cloths					
normal	103	99.0	142	99.3	
too large	0	0.0	1	0.7	0.666
reduced / neglected	1	1.0	0	0.0	
Hair					
Normal (looked after)	104	100	143	100	–
Overall skin					
normal	103	99.0	143	100	0.421
reduced / neglected	1	1.0	0	0.0	
Skin in diaper area					
normal	104	100	141	99.3	1.000
reduced / neglected	0	0.0	1	0.7	
Finger- and toenails					
normal	103	100	141	100	–

^a^ assessed by Fisher's exact test

## Discussion

### Main findings

Our study showed that the 12-month long ‘early support’ program *Babylotse-Plus* had beneficial effects particularly for maternal mental health. The group of mothers receiving the ‘early support’ intervention had a considerably lower occurrence of maternal depression, reported less often a disturbed relationship with their parenting partner, less stress due to the demands of the infant, and more parental competence compared to the parents in the control group. There was no difference in the overall childcare status between the ‘early support group’ and the control group receiving usual routine care. Single childcare and some parenting stress indicators showed no relevant differences between intervention and control group, however none of the indicators suggested any negative effect of the intervention.

### Comparison with other studies

The National Centre on Early Prevention has been set up by the German government to support a number of pilot projects (including our early support program *Babylotse-Plus*) across the country within the framework of the federal action program “Early Prevention and Intervention for Parents and Children and Social Warning Systems”. Explorative evaluations had shown that specifically trained “family midwives” - available for 1 year support - were particularly promising for a successful implementation of early prevention programs [17].

The screening process to determine the psychosocial risk of potential participants for our study was driven by midwives, nursing staff or doctors, whereas the subsequent scoring and - when indicated -the initial intervention was driven by social workers, the so called ‘family/baby pilots’ (“Babylotsen”). These social workers were specifically trained to take the lead in the navigation process to individualized local early support programs for the enrolled families at high psychosocial risk. This approach has been supported by a recent survey among 190 family midwives and nurses in Germany assessing how 937 families with young children benefited from family midwives and nurses. The results suggested positive effects from their support for all families, although those with lower stress and relatively higher resources seemed to benefit more [18]. The available number of these specially trained family midwifes is very low, and a sensible assignment to families with most potential benefit as performed in this study, is very important.

Our early intervention program showed a considerable reduction of postnatal maternal depression, which is a strong stressor in early childhood and is associated with lower levels of sensitive responsive childcare. Maternal depression can negatively affect infant development of emotional regulation skills, interpersonal skills and stress response mechanisms ([10, 19]. Bureau et al showed that maternal depressive symptoms during infancy might lead to the development of depressive symptoms in childhood and adolescence in the offspring even after controlling for other variables of potential relevance [20]. The relevance of these findings was recently confirmed. Young children whose mothers experienced even mild depressive symptoms had increased risk for later behavioral problems, suggesting a possible need for new screening and intervention strategies for mothers with mild or subclinical depression [21].

Our findings indicated improved self-reported parental competence through our ‘early support’ program with only one intervention and up to two follow-ups by telephone in 12 months. This confirmed results of the German study ‘A Good Start in Life’ (‘Guter Start ins Kinderleben’), which examined the Ulm Model intervention including 7 counseling sessions over 3 months at the family’s home by trained health care and youth welfare staff. After the 12-month evaluation of this earlier study, benefits were seen for mothers at high-risk in terms of an improved maternal sensitivity and for children regarding their emotional development [22]. The 3-year follow-up of “A Good Start in Life” showed no beneficial long-term effects of this early parental counseling intervention regarding the children’s development [23]. Our intervention program showed some tendencies but no strong beneficial effects on childcare outcomes. This calls for a stronger focus of future studies to develop interventions that not only improve the mother’s but also the child’s well-being [13, 14].

The positive results of our primary prevention program in families with 1-year old children complement the findings from the SafeCare® project in the USA including home visitations [24]. A statewide, randomized trial in Oklahoma showed its potential as a secondary preventive intervention by significantly reducing child neglect recidivism rates in families with a maltreating parent [25–27]. SafeCare® seems culturally competent and effective in different ethnic groups such as in Native American parents [25, 26]. For our Babylotse-Plus program future studies are still needed that will focus on populations with different ethnic backgrounds.

### Strengths and limitations

It was helpful for the present study to build on valuable previous experiences from the similarly structured predecessor project at the University Medical Center Hamburg-Eppendorf, Germany [28]. Their early support program Babylotse during infancy was modified to the Babylotse-Plus intervention program, which has been implemented in Berlin. The modifications included also the development of a new short perinatal screening form for psychosoical risk detection to be filled out by midwives or nursing staff. The evaluation of the diagnostic accuracy showed that this simple screening instrument can very reliably identify psychosocially burdened families [11].

Several limitations to the study should be noted. Many screened families did not fulfill all our inclusion criteria. The two major reasons for excluding parents were insufficient language skills due to a lack of available translators for the reference standard in our study, i.e. the comprehensive one-hour long interview, and prior involvement in other (mandatory) social and welfare programs – because of underage, domestic violence, severe mental disease or substance abuse. These ongoing programs would have not allowed us to properly determining the possible effects of the newly developed Babylotse-Plus early support program. Therefore, the results of our study cannot be generalized to all parents of newborns who are exposed to high psychosocial burden.

The comparison group was a historical control group, where the screening for a likely psychosocial risk was based only on the review of routine care data. Although we screened a number of different records including the standard mother’s pass (“Mutterpass”) with information throughout the pregnancy, postnatal course and perinatal files, families may have been misclassified. The risk score cut-off value of 3 was purposely chosen rather low to avoid missing families at risk. A preceding separate evaluation showed that the screening form with this low cut-off had a high sensitivity of 99%, meaning that hardly any family at risk was missed. On the contrary, the specificity of the screening form was rather low at 33%, meaning that a number of families were falsely classified as being at risk [11].

Although many families had to be excluded from study participation, we were still able to enroll a relatively large number and broad spectrum of families in terms of maternal age and mental health, socioeconomic and migration status. The statistical power of our study was sufficient to detect overall differences such as reduced maternal depression in the ‘early support’ compared to control group; however, it reached its limits to reliably compare potential subgroup effects of the ‘early support’ intervention e.g. among families with lower and higher social status. According to a recent national survey in Germany, better-educated parents of children aged 0–3 years are generally using individual assistance programs more often than less educated parents. However, family midwife support seems to be particularly more appreciated by families with a lower social status, which is in contrast to the usual perception where better educated families would make more use of prevention services [29]. Such relevant inequalities should be considered in developing early prevention programs and future accompanying scientific evaluations.

Another limitation of our 12-month long study was the loss-to-follow-up in the intervention group. At the end of the observation period at 12 months, we were able to assess almost all participants in the historical control group but only about half of those recruited in the intervention group (Table 2). One reason for that can be that our source population from the hospital’s catchment area was the rather mobile young inner city population of Berlin. We can only speculate about further reasons and need to consider the particularly vulnerable study participants, where some may have been hesitant to return for a second personal assessment on these sensitive issues, which they remembered from the first (baseline) assessment. They may have also considered it as too much stress returning to the hospital in their current situation. Others may have not felt the need for a further assessment if the intervention was successful. We compared those who were lost-to-follow-up with those who stayed in the study and found no considerable difference for the intervention group in terms of baseline risk scores with both median risk scores of 5 and very similar interquartile ranges (Table 2). In both groups, the most common items that contributed to the risk scores were similarly distributed and included (in descending order): mental conditions (especially self-reported depression), migration background, smoking in pregnancy, economic problems, preterm birth, and 2 or more children under 5 years. However, we cannot completely rule out potential bias related to other characteristics that were not assessed.

The child care outcome variables and the mother-child interaction assessment in the present study were chosen as they would indicate signs of neglect and malnutrition. When the mothers were invited for this assessment, they were asked to bring their children with them but they were not specifically informed about the detailed assessment including inspection of the child. We believe that our pediatric nurses who performed these assessments would have identified strong signs related to these outcomes. They would have contacted a pediatrician in case of concern. In our evaluation there was no severe sign for neglect and malnutrition, however we cannot rule out that this would have been the case if those who did not show up would have come. The addition of other measures such as reports or involvement of social services may be considered in future studies on this topic.

## Conclusions

Our results suggest that the 12-month long ‘early support’ program *Babylotse-Plus* may be protective against the development of maternal depression. This may also reduce the risk of developing mental illness in the offspring later in life. Furthermore, the intervention program seemed to help the mothers to gain more (self-reported) competence as a parent and to feel less stressed due to the demands of the child. The program showed no relevant beneficial effects on childcare outcomes. This calls for future studies to focus more on improving not only the well-being of the mother but also of the child.

The systematic approach already performed during pregnancy and around the time of delivery, combined with voluntary, individually tailored supporting programs of specially trained social workers seem to constitute a well-accepted process for preventive child protection and welfare. The offer to participate in the intervention group of our study was refused by only 1% and explained by self-reported lack of time or that the participants felt they had enough support.

Parents want to be “good (enough) parents”. Especially during pregnancy and at around delivery parents seem open to assessment demands, pilot service counseling support and follow-up contacts (e.g. by phone) if such offer has low barriers and does not appear to be stigmatizing. Even parents who may otherwise refuse the help of public institutions like youth welfare seemed to accept appropriate support and opened their doors for home visits by service providers.

Future research is required in different settings including non-academic hospitals and birth centers with more diverse study populations such as families with different ethnic and migration backgrounds. Future studies should also determine long-term effects of the early support program as well as feasibility and benefits of specific support beyond infancy.

## Data Availability

The data for this study is not publicly available but can be provided on request by contacting the corresponding author.

## Abbreviations

95%-CI: 95%-confidence intervals
aOR: Adjusted odds ratios
EBI: Eltern Belastungs Index *[German version of the PSI]*
OR: Odds ratio
PSI: Parenting Stress Index
SAS: Statistical Analysis System Software
UNICEF: United Nations Children’s Fund
WHO: World Health Organization
